# Trends in Respiratory Pathogen Testing at US Children’s Hospitals

**DOI:** 10.1001/jamanetworkopen.2025.0160

**Published:** 2025-03-06

**Authors:** Matthew J. Molloy, Matthew Hall, Jessica L. Markham, Jillian M. Cotter, Elisha McCoy, Michael J. Tchou, Megan E. Collins, Michael J. Steiner, John R. Stephens, Andrew G. Yu, Irma T. Ugalde, Rustin B. Morse, Monika K. Goyal, Samantha A. House

**Affiliations:** 1 Division of Hospital Medicine, Cincinnati Children’s Hospital Medical Center, Cincinnati, Ohio; 2 Department of Pediatrics, University of Cincinnati College of Medicine, Cincinnati, Ohio; 3 Children’s Hospital Association, Lenexa, Kansas; 4 Department of Pediatrics, Children’s Mercy Kansas City, University of Missouri-Kansas City School of Medicine, Kansas City; 5 Department of Pediatrics, University of Kansas School of Medicine, Kansas City; 6 Section of Hospital Medicine, Children’s Hospital Colorado, Aurora; 7 Department of Pediatrics, University of Colorado, Aurora; 8 Department of Pediatrics, Le Bonheur Children’s Hospital, The University of Tennessee Health Science Center, Memphis; 9 Department of Pediatrics, Emory University School of Medicine, Atlanta, Georgia; 10 Children’s Healthcare of Atlanta, Atlanta, Georgia; 11 Department of Pediatrics, University of North Carolina at Chapel Hill; 12 Division of Hospital Medicine, Department of Pediatrics, University of Texas Southwestern Medical Center, Dallas; 13 Children’s Medical Center, Dallas, Texas; 14 Department of Pediatrics, The University of Chicago Pritzker School of Medicine, Chicago, Illinois; 15 Arkansas Children’s Northwest, Springdale; 16 Department of Pediatrics, Children’s National Hospital, The George Washington University, Washington, DC; 17 Department of Pediatrics, Dartmouth Health Children’s, Lebanon, New Hampshire

## Abstract

**Importance:**

Respiratory pathogen testing has been a common deimplementation focus. The COVID-19 pandemic brought new considerations for respiratory testing; recent trends in testing rates are not well understood.

**Objective:**

To measure trends in respiratory testing among encounters for acute respiratory infections among children and adolescents (aged <18 years) from 2016 to 2023, assess the association of COVID-19 with these trends, and describe associated cost trends.

**Design, Setting, and Participants:**

This retrospective serial cross-sectional study included emergency department (ED) encounters and hospitalizations in US children’s hospitals among children and adolescents with a primary acute infectious respiratory illness diagnosis. Data were ascertained from the Pediatric Health Information System database from January 1, 2016, to December 31, 2023.

**Exposure:**

Respiratory pathogen testing.

**Main Outcomes and Measures:**

The primary outcome was the percentage of encounters with respiratory testing over time. Interrupted time series models were created to assess the association of COVID-19 with testing patterns. The inflation-adjusted standardized unit cost associated with respiratory testing was also examined.

**Results:**

There were 5 090 923 eligible encounters among patients who were children or adolescents (mean [SD] age, 3.36 [4.06] years); 55.0% of the patients were male. Among these encounters, 87.5% were ED only, 77.9% involved children younger than 6 years, and 94.5% involved children without complex chronic conditions. Respiratory testing was performed in 37.2% of all encounters. The interrupted time series models demonstrated increasing prepandemic testing rates in both ED-only encounters (slope, 0.26 [95% CI, 0.21-0.30]; *P* < .001) and hospitalizations (slope, 0.12 [95% CI, 0.07-0.16]; *P* < .001). Increases in respiratory testing were seen at the onset of the COVID-19 pandemic in both ED-only encounters (level change, 33.78 [95% CI, 31.77-35.79]; *P* < .001) and hospitalizations (level change, 30.97 [95% CI, 29.21-32.73]; *P* < .001), associated initially with COVID-19–only testing. Postpandemic testing rates remained elevated relative to prepandemic levels. The percentage of encounters with respiratory testing increased from 13.6% [95% CI, 13.5%-13.7%] in 2016 to a peak of 62.2% [95% CI, 62.1%-62.3%] in 2022. While COVID-19–only testing decreased after 2020, other targeted testing and large-panel (>5 targets) testing increased. The inflation-adjusted standardized unit cost associated with respiratory testing increased from $34.2 [95% CI, $33.9-$34.6] per encounter in 2017 to $128.2 [95% CI, $127.7-$128.6] per encounter in 2022.

**Conclusions and Relevance:**

The findings of this cross-sectional study suggest that respiratory testing rates have increased over time, with large increases at the onset of the COVID-19 pandemic that have persisted. Respiratory testing rates and related costs increased significantly, supporting a need for future deimplementation efforts.

## Key Points

**Question:**

How have respiratory pathogen testing rates and costs changed over time for children and adolescents with acute respiratory infections, both before and after the onset of the COVID-19 pandemic?

**Findings:**

In this cross-sectional study of 5 090 923 children’s hospital encounters, respiratory pathogen testing rates were increasing prior to the onset of the COVID-19 pandemic, with large increases at onset that have persisted; after the pandemic, COVID-19–only testing decreased, while large-panel testing continued to increase. Testing costs increased from 2016 to 2023.

**Meaning:**

The findings of this study suggest that the COVID-19 pandemic was associated with increased respiratory pathogen testing rates and costs that have persisted, supporting a need for future deimplementation efforts.

## Teaser

This cross-sectional study assesses whether respiratory pathogen testing rates and costs changed for children and adolescents with acute respiratory infections before and after the onset of the COVID-19 pandemic.

## Introduction

Respiratory tract infections are among the most common reasons for pediatric emergency department (ED) or inpatient care, accounting for a significant proportion of hospital-based resource utilization.^1,2,3,4^ While these infections can be diagnosed based on presenting clinical features, diagnostic tests (typically nasopharyngeal swabs) are still commonly used to identify causative pathogens.^5,6,7^ Reducing respiratory pathogen testing has been a common deimplementation focus given associated costs and the limited association with clinical management decisions in many clinical scenarios.^5,6^

While deimplementation initiatives have decreased respiratory pathogen testing for some pediatric patients over recent decades,^5,6,8,9,10^ the COVID-19 pandemic brought with it new considerations surrounding the implications of such tests. Utilization of tests to detect the novel SARS-CoV-2 virus was an important public health tool given the unique isolation approaches used to reduce disease spread. Additionally, there was renewed interest in characterizing the epidemiology of viruses during and after the pandemic given observed disruptions to usual seasonal viral patterns.^11,12,13^ COVID-19 testing has been associated with a perceived increase in hospital-based respiratory pathogen testing since 2020. While evidence supports this pattern for some diagnoses,^14^ studies describing current respiratory testing patterns and associated costs across respiratory conditions are lacking. Data describing these patterns may inform deimplementation efforts in the postpandemic era of pediatric care.

In this study, we aimed to measure trends in respiratory pathogen testing rates among children and adolescents with acute respiratory infections at US children’s hospitals from 2016 to 2023, assess the association of the COVID-19 pandemic with these trends, and describe cost trends associated with respiratory testing.

## Methods

### Study Design and Data Source

We conducted a retrospective serial cross-sectional study using the Pediatric Health Information System (PHIS), an administrative database maintained by the Children’s Hospital Association containing patient demographics, billing data, and diagnosis and procedure codes (using *International Statistical Classification of Diseases and Related Health Problems, Tenth Revision* [*ICD-10*]) from 47 children’s hospitals in the US. The Children’s Hospital Association and participating hospitals jointly ensure data quality. We included data from 38 hospitals, excluding 9 hospitals that did not contribute data for the complete study period. Use of deidentified PHIS data is not considered human participant research by the Cincinnati Children’s Hospital Medical Center Institutional Review Board, and thus informed consent was waived. The study followed the Strengthening the Reporting of Observational Studies in Epidemiology (STROBE) reporting guideline.

### Study Population

We included ED-only encounters and hospitalizations (inpatient and observation) for children and adolescents (aged <18 years) with a primary diagnosis of an acute infectious respiratory illness from January 1, 2016, to December 31, 2023. To identify included encounters, we first examined Pediatric Clinical Classification System categories, which classify groups of *ICD-10* codes into mutually exclusive, clinically meaningful pediatric conditions,^15^ identifying categories potentially containing acute infectious respiratory illness. We then evaluated all *ICD-10* codes within each selected Pediatric Clinical Classification System category and included codes consistent with an acute infectious respiratory illnesses based on group consensus to derive a list of included *ICD-10* codes (eTable 1 in Supplement 1). To minimize the risk of misclassification bias, we excluded encounters in which patients were transferred in or out of the hospital that submitted data, as respiratory testing obtained at another hospital would not be captured in these cases.

To evaluate potential differences in respiratory pathogen testing rates by setting, encounters were stratified into 3 types based on the highest level of care provided during the encounter: (1) ED only, (2) hospitalizations without intensive care unit (ICU) visits, and (3) hospitalizations with ICU visits.

### Patient Characteristics

We examined patient demographics, including age, sex, race and ethnicity, primary payer, and Child Opportunity Index (COI).^16^ Race and ethnicity were examined as social constructs and were included in our analysis due to previously reported racial and ethnic disparities in bronchiolitis diagnostic testing practices and rates of SARS-CoV-2 infection.^17,18^ Race and ethnicity data were collected according to hospital-specific practices and mapped to PHIS categories. We included race and ethnicity categories of Asian, Hispanic, non-Hispanic Black, non-Hispanic White, and other (including American Indian or Alaska Native, Native Hawaiian or Other Pacific Islander, other, multiracial, and missing). The COI is a measure of neighborhood-level resource availability based on the patient’s residential zip code; a lower COI has been associated with a higher hospitalization burden for respiratory illness.^19^ Additionally, we examined the number of complex chronic conditions (CCCs)^20^ and the presence of either a respiratory or technology-dependent CCC, as respiratory testing patterns may differ based on a patient’s known chronic conditions. We assessed severity of illness using hospitalization resource intensity scores for kids (H-RISK), a pediatric-specific measure of illness severity and resource utilization.^21^

### Respiratory Pathogen Testing

We identified respiratory pathogen testing through the index list of billing clinical transaction classification codes in the PHIS database. We used a list of respiratory testing codes developed and validated by Shapiro et al^22^ and expanded this list to include clinical transaction classification codes for SARS-CoV-2 (COVID-19) testing (eTable 2 in Supplement 1). To further characterize testing patterns, we categorized each clinical transaction classification code into 3 mutually exclusive categories: (1) COVID-19–only testing (ie, testing for the SARS-CoV-2 virus alone), (2) targeted respiratory testing (≤5 targets with or without SARS-CoV-2), and (3) large-panel respiratory testing (>5 targets with or without SARS-CoV-2). We chose 5 or fewer targets as the threshold for targeted testing through author consensus, as it was believed to delineate common tests evaluating for SARS-CoV-2, influenza A/B viruses, and respiratory syncytial viruses from larger respiratory testing panels.

### Outcome Measures

Our primary outcome was the percentage of encounters with any respiratory pathogen testing. We assessed the percentage of encounters with respiratory testing in aggregate and by encounter type (ED only, hospitalization without ICU visit, and hospitalization with ICU visit); testing rates were measured at quarterly intervals. We also evaluated the inflation-adjusted standardized unit cost associated with respiratory pathogen testing, both in aggregate (ie, total cost of respiratory testing) and per encounter. Standardized unit cost was developed by the Children’s Hospital Association to compare resource utilization across hospitals in the setting of interhospital variation in cost.^23^ Therefore, standardized unit cost does not directly measure individual hospital spending but rather serves as a measure of relative resource-use intensity applicable across PHIS hospitals. Respiratory pathogen testing cost per encounter was calculated by dividing the total cost of respiratory testing by the number of encounters for the relevant period.

### Statistical Analysis

We performed unadjusted comparisons of patient and encounter characteristics among encounters with and without respiratory pathogen testing using χ^2^ tests. We modeled trends in the percentage of encounters with respiratory pathogen testing and in the inflation-adjusted standardized unit cost associated with respiratory pathogen testing per encounter by year using general estimating equations clustered on hospital.

To assess the association of the COVID-19 pandemic with respiratory pathogen testing patterns, we modeled temporal change in monthly testing rates using interrupted time series models adjusting for hospital, seasonality, age, number of CCCs, and H-RISK. We created 2 interrupted time series analyses: ED only and hospitalizations. For both models, we used the onset of the COVID-19 pandemic in March 2020 to define the segments before and after onset and censored March and April 2020 given evolving availability of COVID-19 testing over these months. All statistical analyses were performed using SAS, version 9.4 (SAS Institute Inc), and a 2-sided *P* < .05 was considered statistically significant.

## Results

### Patient and Encounter Characteristics

We identified 5 090 923 encounters with children and adolescents (mean [SD] age, 3.36 [4.06] years) for acute, infectious respiratory illness from 2016 to 2023; 45.0% of the patients were female and 55.0% were male. Of these encounters, 87.5% were ED only, 77.9% were among children younger than 6 years, 35.0% were among children in the lowest COI quintile, and 94.5% were among children without CCCs (Table 1). Of the 634 303 hospitalizations, 22.1% required an ICU visit.

**Table 1.** Patient and Encounter Characteristics of Encounters for Acute, Infectious Respiratory Illness With and Without Respiratory Pathogen Testing^a^

Characteristic	Total encounters, No. (%)^b^	Encounter, No. (%)^c^	
		Without respiratory test	With respiratory test
Encounters, No.	5 090 923 (100.0)	3 196 767 (62.8)	1 894 156 (37.2)
Age, y			
<2	2 390 617 (47.0)	1 530 234 (64.0)	860 383 (36.0)
3-5	1 572 620 (30.9)	1 015 150 (64.6)	557 470 (35.4)
6-12	857 966 (16.9)	507 465 (59.1)	350 501 (40.9)
13-17	269 720 (5.3)	143 918 (53.4)	125 802 (46.6)
Sex			
Female	2 292 971 (45.0)	1 435 673 (62.6)	857 298 (37.4)
Male	2 797 669 (55.0)	1 760 946 (62.9)	1 036 723 (37.1)
Race and ethnicity			
Asian	170 147 (3.3)	102 782 (60.4)	67 365 (39.6)
Hispanic	1 643 891 (32.3)	1 034 053 (62.9)	609 838 (37.1)
Non-Hispanic Black	1 293 088 (25.4)	803 931 (62.2)	489 157 (37.8)
Non-Hispanic White	1 599 700 (31.4)	1 009 325 (63.1)	590 375 (36.9)
Other^d^	384 097 (7.5)	246 676 (64.2)	137 421 (35.8)
Payer			
Government	3 434 834 (67.5)	2 159 397 (62.9)	1 275 437 (37.1)
Private	1 278 676 (25.1)	780 397 (61.0)	498 279 (39.0)
Other	377 413 (7.4)	256 973 (68.1)	120 440 (31.9)
Child Opportunity Index^e^			
Very low	1 779 592 (35.0)	1 167 780 (65.6)	611 812 (34.4)
Low	981 803 (19.3)	621 472 (63.3)	360 331 (36.7)
Moderate	839 026 (16.5)	507 037 (60.4)	331 989 (39.6)
High	805 951 (15.9)	491 219 (60.9)	314 732 (39.1)
Very high	671 233 (13.2)	399 855 (59.6)	271 378 (40.4)
No. of complex chronic conditions^20^			
0	4 811 312 (94.5)	3 101 193 (64.5)	1 710 119 (35.5)
1	170 551 (3.4)	71 260 (41.8)	99 291 (58.2)
≥2	109 060 (2.1)	24 314 (22.3)	84 746 (77.7)
Complex chronic condition category			
Respiratory	54 781 (1.1)	16 885 (30.8)	37 896 (69.2)
Technology dependence	105 396 (2.1)	26 246 (24.9)	79 150 (75.1)
Encounter type			
ED only	4 456 620 (87.5)	2 978 448 (66.8)	1 478 172 (33.2)
Hospitalization without ICU visit	494 413 (9.7)	194 817 (39.4)	299 596 (60.6)
Hospitalization with ICU visit	139 890 (2.7)	23 502 (16.8)	116 388 (83.2)

Abbreviations: ED, emergency department; ICU, intensive care unit.

^a^ All comparisons were significant (*P* < .001).

^b^ The percentages (column percentages) are derived from the total number of encounters for the entire cohort.

^c^ The percentages (row percentages) are derived from the total number of encounters for a given characteristic.

^d^ Includes American Indian or Alaska Native, Native Hawaiian or Other Pacific Islander, other, multiracial, and missing.

^e^ Based on patient’s residential zip code to measure neighborhood-level resource availability.

### Respiratory Pathogen Testing

Across the study period, 37.2% of encounters had respiratory pathogen testing. Hospitalizations with ICU visits had the highest percentage of respiratory testing (83.2% of encounters), followed by hospitalizations without ICU visits (60.6%), and ED-only encounters (33.2%). Unadjusted comparisons between encounters with and without respiratory pathogen testing are shown in Table 1. All comparisons in Table 1 were significant. Younger patients (aged ≤5 years) were less likely to have respiratory pathogen testing than were older patients (aged >5 years). Respiratory testing rates were greater in the highest COI quintile (40.4%) compared with the lowest COI quintile (34.4%). Patients with more CCCs were more likely to receive respiratory pathogen testing (35.5% for 0 CCCs vs 77.7% for 2 or more CCCs).

### Trends in Respiratory Pathogen Testing Over Time

**Figure 1.**
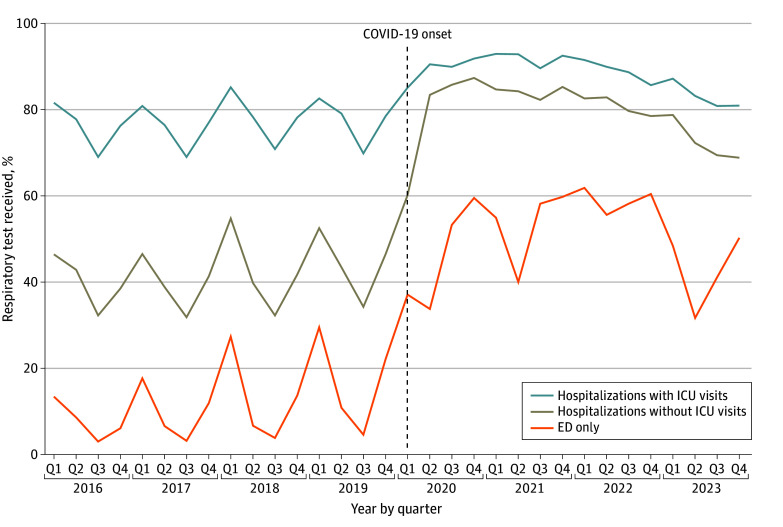
Unadjusted Percentage of Encounters With Any Respiratory Pathogen Testing, 2016-2023 ED indicates emergency department; ICU, intensive care unit; Q, quarter.

Figure 1 demonstrates unadjusted respiratory pathogen testing rates over time by encounter type. Prior to the onset of the COVID-19 pandemic, increasing rates of respiratory testing marked by distinct seasonality were observed across all settings, with the highest rates of testing in January to March and the lowest rates in July to September of each year. At the onset of the pandemic, we observed increases in respiratory pathogen testing in all settings, with decreased seasonal variation. Thereafter, testing rates remained elevated relative to pre–COVID-19 levels. Seasonal patterns appeared to reemerge in 2023, particularly in ED-only encounters. When adjusting testing rates for hospital clustering, we observed a significant increase in testing overall and for each encounter type over the study period (Table 2). The percentage of all encounters with respiratory pathogen testing increased from 13.6% [95% CI, 13.5%-13.7%] in 2016 to a peak of 62.2% [95% CI, 62.1%-62.3%] in 2022. The largest increase was seen in ED-only encounters (from 8.8% [95% CI, 8.7%-8.9%] in 2016 to 59.3% [95% CI, 59.2%-59.4%] in 2022). The smallest increase was seen in hospitalizations with ICU visits (from 77.2% [95% CI, 76.6%-77.9%] in 2017 to 91.7% [95% CI, 91.3%-92.1%] in 2021); this group demonstrated the highest testing rates at the start of the study period.

**Table 2.** Trends in Respiratory Pathogen Testing and Cost of Respiratory Pathogen Testing, 2016-2023^a^

Encounter type and year	No. of encounters	Encounters with respiratory testing, No. (% [95% CI])	*P* value for test of trend in % of encounters with testing	Cost of respiratory testing		
				Total, million $	Per encounter	
					Mean (95% CI), $	*P* value for test of trend
**Total encounters**						
2016	586 900	79 914 (13.6 [13.5-13.7])	<.001	20.6	35.1 (34.6-35.5)	<.001
2017	631 616	102 602 (16.2 [16.2-16.3])		21.6	34.2 (33.9-34.6)	
2018	649 639	136 769 (21.1 [21-21.2])		26.3	40.5 (40.1-40.9)	
2019	690 690	170 261 (24.7 [24.5-24.8])		33.2	48.1 (47.8-48.5)	
2020	375 119	173 915 (46.4 [46.2-46.5])		28.6	76.2 (75.6-76.8)	
2021	624 425	366 796 (58.7 [58.6-58.9])		60.9	97.5 (97.0-98.0)	
2022	866 569	539 073 (62.2 [62.1-62.3])		111.0	128.2 (127.7-128.6)	
2023	665 965	324 826 (48.8 [48.7-48.9])		83.2	124.9 (124.3-125.6)	
**ED only**						
2016	515 424	45 222 (8.8 [8.7-8.9])	<.001	6.4	12.4 (12.2-12.6)	<.001
2017	555 947	65 456 (11.8 [11.7-11.9])		7.6	13.7 (13.5-13.9)	
2018	571 815	95 776 (16.7 [16.7-16.8])		10.9	19.1 (18.9-19.3)	
2019	602 349	122 772 (20.4 [20.3-20.5])		16.0	26.5 (26.2-26.7)	
2020	331 073	141 648 (42.8 [42.6-43])		17.5	52.8 (52.4-53.2)	
2021	549 520	302 438 (55 [54.9-55.2])		40.6	74 (73.6-74.3)	
2022	757 304	449 344 (59.3 [59.2-59.4])		80.4	106.1 (105.8-106.5)	
2023	573 188	255 516 (44.6 [44.4-44.7])		55.0	96.0 (95.6-96.5)	
**Hospitalization without ICU visit**						
2016	57 743	24 040 (41.6 [41.2-42.0])	<.001	8.7	153.5 (151.0-156.1)	<.001
2017	59 668	24 793 (41.6 [41.2-41.9])		8.4	141.2 (139.1-143.3)	
2018	60 772	27 407 (45.1 [44.7-45.5])		8.9	147.0 (144.8-149.3)	
2019	68 161	31 592 (46.3 [46.0-46.7])		9.9	144.6 (142.7-146.4)	
2020	33 159	22 750 (68.6 [68.1-69.1])		6.8	205.7 (202.6-208.8)	
2021	56 715	47 675 (84.1 [83.8-84.4])		13.0	228.9 (226.5-231.3)	
2022	85 563	68 788 (80.4 [80.1-80.7])		20.7	242.2 (240.1-244.3)	
2023	72 632	52 551 (72.4 (72.0-72.7])		19.0	261.5 (258.1-265.0)	
**Hospitalization with ICU visit**						
2016	13 733	10 652 (77.6 [76.9-78.3])	.004	5.3	388.2 (375.1-401.3)	.09
2017	16 001	12 353 (77.2 [76.6-77.9])		5.6	347.3 (340.2-354.3)	
2018	17 052	13 586 (79.7 [79.1-80.3])		6.4	376.9 (369.3-384.5)	
2019	20 180	15 897 (78.8 [78.2-79.3])		7.4	368.6 (361.6-375.6)	
2020	10 887	9517 (87.4 [86.8-88.0])		4.4	393.8 (383.3-404.2)	
2021	18 190	16 683 (91.7 [91.3-92.1])		7.2	397.7 (388.3-407.1)	
2022	23 702	20 941 (88.4 [87.9-88.8])		9.6	420.3 (412.1-428.5)	
2023	20 145	16 759 (83.2 [82.7-83.7])		9.2	455.2 (444.5-465.9)	

Abbreviations: ED, emergency department; ICU, intensive care unit.

^a^ Trends were tested using generalized estimating equations clustered on hospital. Costs are inflation-adjusted standardized unit costs. Total cost is shown as millions of dollars, rounded to the nearest hundred-thousand dollars.

**Figure 2.**
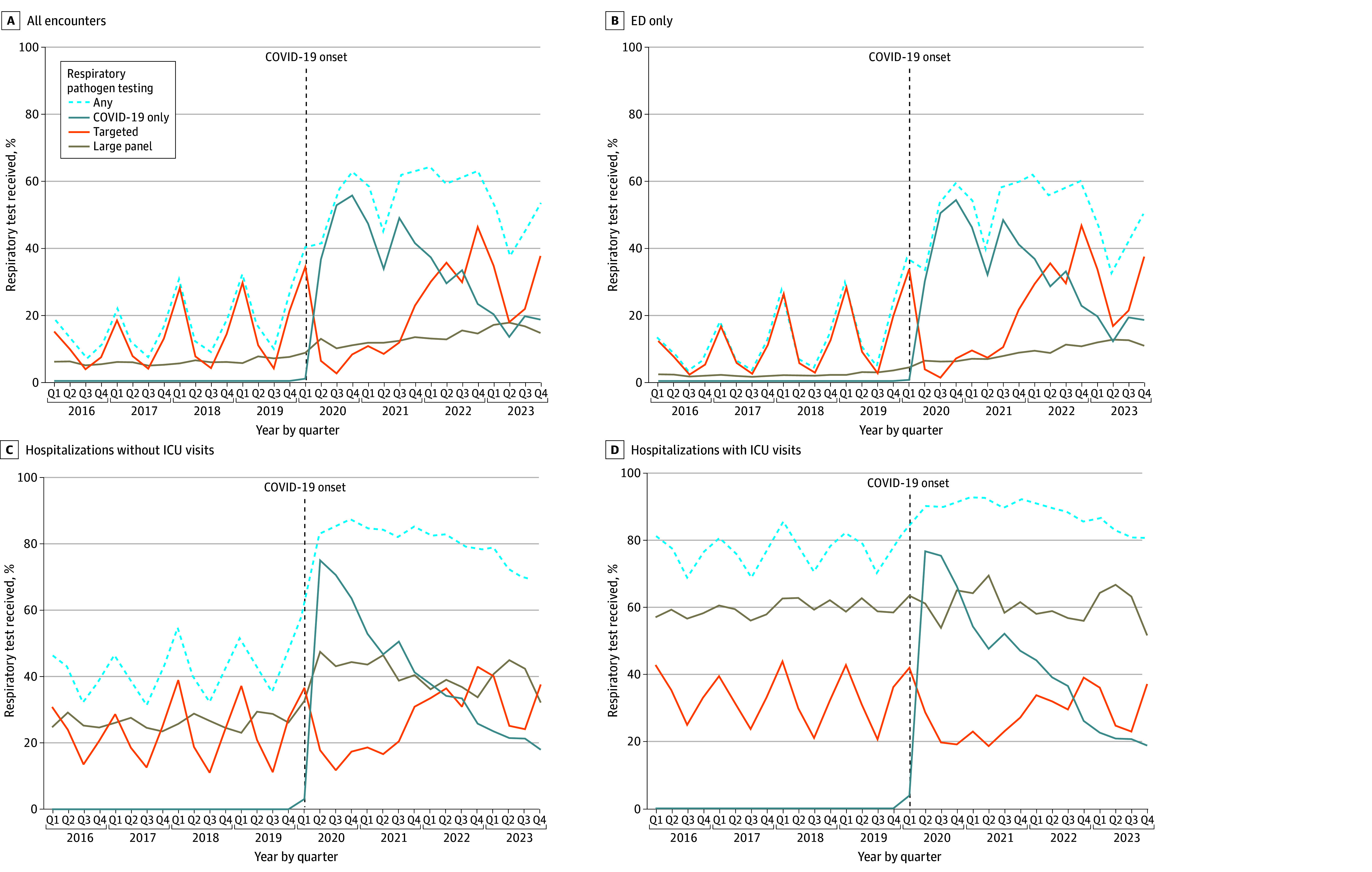
Time Series of Unadjusted Percentage of Encounters With Categories of Respiratory Pathogen Testing, 2016-2023 Targeted respiratory testing is 5 or fewer targets; large-panel respiratory testing is more than 5 targets. ED indicates emergency department; ICU, intensive care unit; Q, quarter.

Figure 2 demonstrates unadjusted respiratory pathogen testing rates by testing category, overall and stratified by encounter type. COVID-19–only testing predominated the overall increase in testing observed in early 2020 in all settings. After 2020, COVID-19–only testing gradually decreased and was present in fewer than 20% of included encounters by the conclusion of the study period. However, over this same period, increases in other forms of testing were associated with overall testing levels that were maintained above those observed prior to the pandemic. Large-panel testing demonstrated a continued gradual increase over the post–COVID-19 period in the ED setting, suggesting an overall increase in this testing modality, while targeted testing patterns were more variable during this period.

**Figure 3.**
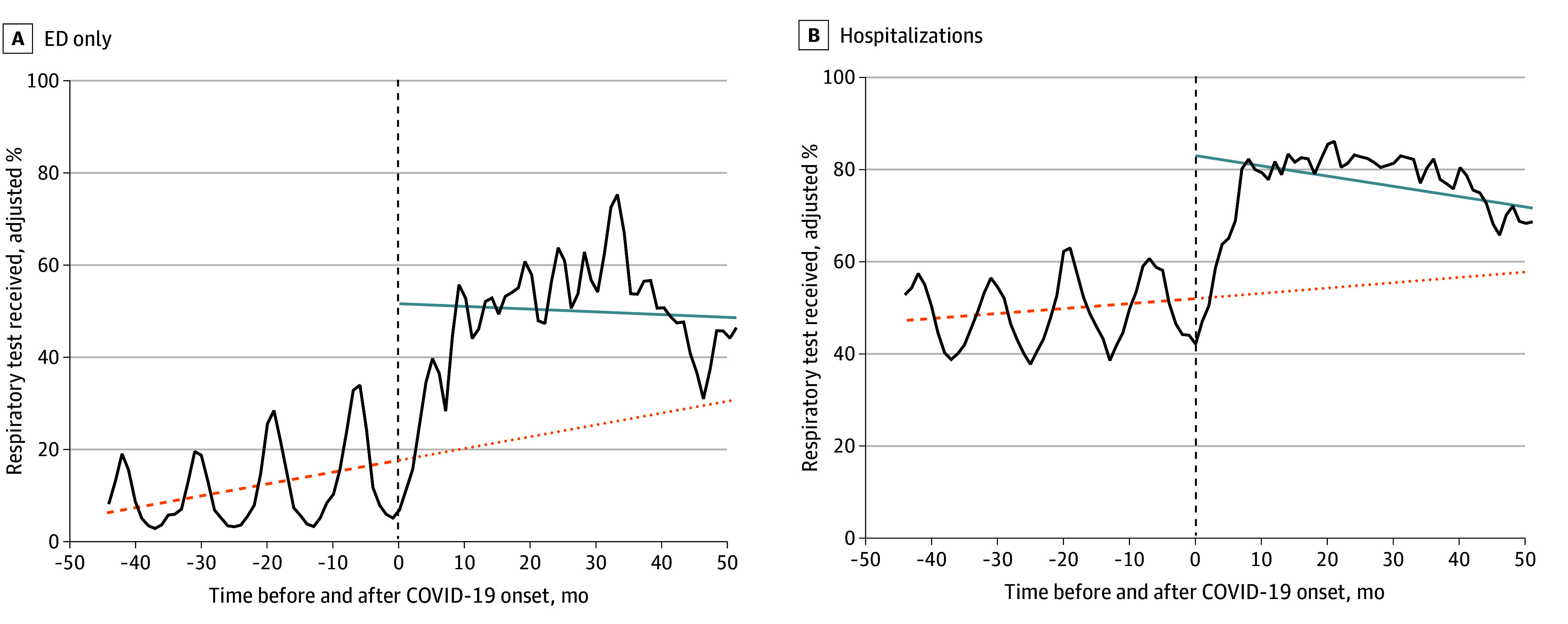
Interrupted Time Series Model Analysis of Adjusted Percentage of Encounters With Respiratory Pathogen Testing The onset of the COVID-19 pandemic in March 2020 (dashed vertical line) defines the segments before and after COVID-19; the months of March and April 2020 were censored. A, Model for emergency department (ED)-only encounters adjusting for hospital, seasonality, age, and number of complex chronic conditions.^20^ Prepandemic slope: 0.26 (95% CI, 0.21-0.30); level change at COVID-19 onset: 33.78 (95% CI, 31.77-35.79); and change in slope from before to after the pandemic: −0.32 (95% CI, −0.39 to −0.24) (*P* < .001). B, Model for hospitalizations adjusting for hospital, seasonality, age, number of complex chronic conditions,^20^ and hospitalization resource intensity scores for kids.^21^ Prepandemic slope: 0.12 (95% CI, 0.07-0.16); level change at COVID-19 onset: 30.97 (95% CI, 29.21-32.73); and change in slope from before to after the pandemic: −0.34 (95% CI, −0.40 to −0.28) (*P* < .001). The orange dashed line indicates the slope of respiratory pathogen testing before the onset of the COVID-19 pandemic; the orange dotted line is a counterfactual trend line (extrapolation of the prepandemic slope). The light blue solid line indicates the slope of respiratory pathogen testing after the onset of the COVID-19 pandemic. The solid black line indicates the adjusted percentage of respiratory tests received.

Figure 3 represents interrupted time series analyses assessing the association of the COVID-19 pandemic with adjusted respiratory testing rates. During the prepandemic period, there was a positive slope in respiratory testing rates in ED-only encounters (0.26 [95% CI, 0.21-0.30]; *P* < .001) and in hospitalizations (0.12 [95% CI, 0.07-0.16]; *P* < .001). At the onset of the pandemic, there was a significant immediate increase in the percentage of encounters with respiratory pathogen testing in both ED-only encounters (level change, 33.78 [95% CI, 31.77-35.79]; *P* < .001) and hospitalizations (level change, 30.97 [95% CI, 29.21-32.73]; *P* < .001). After the onset of the pandemic, there was a significant decrease in slope in both ED-only encounters (−0.32 [95% CI, −0.39 to −0.24]; *P* < .001) and hospitalizations (−0.34 [95% CI, −0.40 to −0.28]; *P* < .001), although rates remained well above pre-pandemic rates.

### Cost of Respiratory Pathogen Testing Over Time

We observed an increase in inflation-adjusted standardized unit cost associated with respiratory pathogen testing over the study period, from a low of $20.6 million in 2016 to a high of $111.0 million in 2022 (Table 2). In models of inflation-adjusted standardized unit cost of respiratory testing per encounter over time clustered on hospital, there was an increase in adjusted cost per encounter, from a low of $34.2 (95% CI, $33.9-$34.6; *P* < .001) per encounter in 2017 to a high of $128.2 (95% CI, $127.7-$128.6; *P* < .001) per encounter in 2022. Statistically significant increases in adjusted cost per encounter occurred in ED-only encounters ($12.4 [95% CI, $12.2-$12.6] in 2016 vs $106.1 [95% CI, $105.8-$106.5] in 2022; *P* < .001) and in hospitalizations without ICU visits ($141.2 [95% CI, $139.1-$143.3] in 2017 vs $261.5 [95% CI, $258.1-$265.0] in 2023; *P* < .001). The adjusted cost of respiratory testing per encounter did not change in hospitalizations with ICU visits.

## Discussion

In this retrospective cross-sectional study of respiratory pathogen testing trends from 2016 to 2023 across 38 children’s hospitals, we observed increasing testing rates with the onset of the COVID-19 pandemic that have not returned to prepandemic levels, with testing rates increasing from 13.6% in 2016 to 62.2% in 2022. Tests assessing for COVID-19 only increased rapidly after their introduction but have decreased in the postpandemic period, with current testing comprised largely of multitarget panels. The increased respiratory pathogen testing observed in this study was associated with an increase from $20.6 million in 2016 to $111.0 million in 2022 in testing-related costs. Prior seasonal trends in respiratory testing were disrupted by the pandemic, with some early evidence of return near the conclusion of our study period.

Contrary to prior literature assessing trends in respiratory testing, our analysis identified increasing testing rates in the prepandemic period in both ED and inpatient settings.^5^ While our study population is broader than some enrolled in prior studies, these findings may suggest challenges with sustaining improvements achieved through deimplementation initiatives. We hypothesize multiple potential drivers for testing trends observed prior to the pandemic. Increased availability of rapid, point-of-care respiratory viral testing may have driven increased testing, particularly in the ED setting.^24^ This increase in availability has also lowered the cost of some testing modalities. Prepandemic qualitative analyses exploring clinician perspectives around respiratory pathogen testing in bronchiolitis identified some challenges to deimplementation efforts, including reassurance provided by definitive diagnosis and perceptions that peers, other clinicians, or families desire testing.^25,26^ The COVID-19 pandemic may have altered both clinician and parental perceptions about the value of testing to establish a diagnosis. Finally, respiratory pathogen testing may be perceived as a relatively low-harm service, which may reduce motivation to prioritize this service for deimplementation.

The sharp increase in respiratory pathogen testing observed at the onset of the COVID-19 pandemic was associated with specific testing for the SARS-CoV-2 virus. Testing to diagnose COVID-19 was a critical public health tool to prevent disease spread, and many health systems had infection-control policies to test even asymptomatic patients during the early pandemic.^27,28^ As the pandemic progressed, COVID-19–only testing was supplanted by other targeted testing (≤5 targets) and large-panel testing (>5 targets). Many of these targeted panels include SARS-CoV-2 along with other common viral targets (ie, influenza viruses and/or respiratory syncytial viruses). However, a limitation of the dataset is a lack of granular information about the targets of each multitarget test. We suspect that the rise in targeted testing seen in late 2021 represents the use of 3 to 5 target respiratory pathogen tests that included SARS-CoV-2.

Targeted testing for SARS-CoV-2 and influenza viruses may have public health implications and can alter management decisions in higher-risk or acuity populations, guiding clinicians in the use of antivirals and other recommended therapies.^29,30^ Yet, the overall value of respiratory testing remains uncertain, and testing for other targets, such as respiratory syncytial virus, may not add value. National guidelines recommend against routine respiratory testing in many pediatric patient populations.^31,32^ Respiratory pathogen testing has not been associated with improved patient outcomes, including lack of reduction in antibiotic use.^22,33,34^ The continued gradual increase of large-panel testing over the study period, in particular, may represent overuse of a costly, low-value intervention. Given the significant increase in the cost of testing per encounter over the study period (eg, from $34.2 in 2017 to $128.2 in 2022), large-panel testing presents an opportunity for targeted deimplementation efforts, especially in the ED, where testing results may not be available prior to patient disposition. Prepandemic deimplementation efforts targeting large-panel testing have been successful even in the pediatric ICU setting.^35^ Given increased availability of over-the-counter testing,^36^ there may also be an opportunity to shift decision-making around testing that will not influence clinical management from the health care system to the family, potentially decreasing both the number of visits and associated costs.

Our results also suggest that there are disparities in rates of respiratory pathogen testing by neighborhood opportunity, with higher rates of respiratory pathogen testing among encounters associated with the highest opportunity neighborhoods. This mirrors a recent study identifying an association between increased delivery of low-value care and higher neighborhood opportunity.^37^ The finding is also notable in the context of known socioeconomic disparities in COVID-19 incidence, severity of illness, and outcomes.^38,39^ More research is needed to understand disparate testing patterns across pediatric patient populations.

### Limitations

Our findings should be interpreted in the context of some limitations. The PHIS database is an administrative dataset with limited ability to assess nuanced clinical factors that may have influenced testing. As a result, we cannot comment on the appropriateness of testing conducted in individual clinical encounters. Our analysis was limited to encounters with a primary diagnosis of acute infectious respiratory illness; we did not explore testing rates among encounters with a primary diagnosis of chronic respiratory illnesses, such as asthma. Additionally, we did not directly explore testing patterns of patients with nonrespiratory symptoms during the COVID-19 pandemic, which were frequently indicated by hospital policies; notably, some patients with positive surveillance testing and without respiratory symptoms may have been included within this cohort, owing to reliance on discharge diagnoses, which may have been influenced by testing results. While we excluded transfers from other hospitals, we cannot ascertain whether prior testing in another health care setting or at home may have influenced testing decisions in individual encounters.

Our analysis did not examine associated changes in outcomes (eg, length of stay, readmission rate) or other practice changes (eg, trends in other laboratory testing). The data are limited to 38 children’s hospitals, which may limit generalizability of our findings to other clinical settings. Additionally, these data only reflect hospital-based care and do not capture trends in ambulatory settings, where increased availability of point-of-care testing may also have contributed to important changes in respiratory pathogen testing rates. Given the nature of the standardized unit-cost measure, our findings around testing-related cost should be interpreted as a reflection of relative cost intensity rather than true direct costs of services. We also did not explore relative cost contributions of different testing categories; future work in this area may inform value-based deimplementation strategies.

## Conclusions

In this cross-sectional study of respiratory pathogen testing trends at US children’s hospitals over 8 years, increasing rates of testing, with large increases at the onset of the COVID-19 pandemic that have persisted, were identified. Respiratory pathogen testing rates and associated costs both increased significantly over the study period, supporting a need for future value-oriented deimplementation efforts.

## Notes

Supplement 1. **eTable 1.** Diagnosis Codes for Acute, Infectious Respiratory Illness **eTable 2.** Respiratory Pathogen Testing Codes and Categories

Supplement 2.
Data Sharing Statement
