# Supplementary material for: Trends in Respiratory Pathogen Testing at US Children’s Hospitals
Source: JAMA Netw Open. 2025 Mar 6;8(3):e250160. doi: 10.1001/jamanetworkopen.2025.0160 (PMC11886727; doi:10.1001/jamanetworkopen.2025.0160)
Supplement: Supplement 1. — eTable 1. Diagnosis Codes for Acute, Infectious Respiratory Illness eTable 2. Respiratory Pathogen Testing Codes and Categories [file jamanetwopen-e250160-s001.pdf]

Supplemental Online Content

Molloy MJ, Hall M, Markham JL, et al. Trends in respiratory pathogen testing at US children's hospitals. *JAMA Netw Open*. 2025;8(3):e250160. doi:10.1001/jamanetworkopen.2025.0160

eTable 1. Diagnosis Codes for Acute, Infectious Respiratory Illness
eTable 2. Respiratory Pathogen Testing Codes and Categories

This supplemental material has been provided by the authors to give readers additional information about their work.

© 2025 Molloy MJ et al. *JAMA Network Open*.

eTable 1. Diagnosis Codes for Acute, Infectious Respiratory Illness

ICD-10	Diagnosis	PECCS Category Description
J210	Acute bronchiolitis due to respiratory syncytial virus	Acute bronchiolitis
J211	Acute bronchiolitis due to human metapneumovirus	Acute bronchiolitis
J218	Acute bronchiolitis due to other specified organisms	Acute bronchiolitis
J219	Acute bronchiolitis, unspecified	Acute bronchiolitis
J200	Acute bronchitis due to Mycoplasma pneumoniae	Acute bronchitis
J201	Acute bronchitis due to Hemophilus influenzae	Acute bronchitis
J202	Acute bronchitis due to streptococcus	Acute bronchitis
J203	Acute bronchitis due to coxsackievirus	Acute bronchitis
J204	Acute bronchitis due to parainfluenza virus	Acute bronchitis
J205	Acute bronchitis due to respiratory syncytial virus	Acute bronchitis
J206	Acute bronchitis due to rhinovirus	Acute bronchitis
J207	Acute bronchitis due to echovirus	Acute bronchitis
J208	Acute bronchitis due to other specified organisms	Acute bronchitis
J209	Acute bronchitis, unspecified	Acute bronchitis
J040	Acute laryngitis	Acute upper respiratory infection
J0430	Supraglottitis, unspecified, without obstruction	Acute upper respiratory infection
J0431	Supraglottitis, unspecified, with obstruction	Acute upper respiratory infection
J060	Acute laryngopharyngitis	Acute upper respiratory infection
J069	Acute upper respiratory infection, unspecified	Acute upper respiratory infection
J40	Bronchitis, not specified as acute or chronic	Bronchitis, not specified as acute or chronic
J00	Acute nasopharyngitis [common cold]	Common cold
R05	Cough	Cough
R051	Acute cough	Cough
R052	Subacute cough	Cough
R058	Other specified cough	Cough
R059	Cough, unspecified	Cough
J042	Acute laryngotracheitis	Croup
J050	Acute obstructive laryngitis [croup]	Croup
R0600	Dyspnea, unspecified	Dyspnea
R0609	Other forms of dyspnea	Dyspnea
J860	Pyothorax with fistula	Empyema/pyothorax
J869	Pyothorax without fistula	Empyema/pyothorax
J09X2	Flu due to ident novel influenza A virus w oth resp manifest	Influenza
J09X9	Flu due to ident novel influenza A virus w oth manifest	Influenza
J101	Flu due to oth ident influenza virus w oth resp manifest	Influenza
J1089	Influenza due to oth ident influenza virus w oth manifest	Influenza

© 2025 Molloy MJ et al. *JAMA Network Open*.

J111	Flu due to unidentified influenza virus w oth resp manifest	Influenza
J1183	Influenza due to unidentified influenza virus w otitis media	Influenza
J1189	Influenza due to unidentified influenza virus w oth manifest	Influenza
J22	Unspecified acute lower respiratory infection	Other lower respiratory disease
R0602	Shortness of breath	Other lower respiratory disease
R0603	Acute respiratory distress	Other lower respiratory disease
R0682	Tachypnea, not elsewhere classified	Other lower respiratory disease
R0689	Other abnormalities of breathing	Other lower respiratory disease
R069	Unspecified abnormalities of breathing	Other lower respiratory disease
J399	Disease of upper respiratory tract, unspecified	Other upper respiratory disease
J9801	Acute bronchospasm	Other upper respiratory disease
R067	Sneezing	Other upper respiratory disease
R070	Pain in throat	Other upper respiratory disease
R0981	Nasal congestion	Other upper respiratory disease
A3700	Whooping cough due to Bordetella pertussis without pneumonia	Pertussis
A3710	Whooping cough due to Bordetella parapertussis w/o pneumonia	Pertussis
A3780	Whooping cough due to other Bordetella species w/o pneumonia	Pertussis
A3790	Whooping cough, unspecified species without pneumonia	Pertussis
J90	Pleural effusion, not elsewhere classified	Pleural effusion
J918	Pleural effusion in other conditions classified elsewhere	Pleural effusion
A3701	Whooping cough due to Bordetella pertussis with pneumonia	Pneumonia
A3711	Whooping cough due to Bordetella parapertussis w pneumonia	Pneumonia
A3781	Whooping cough due to oth Bordetella species with pneumonia	Pneumonia
A3791	Whooping cough, unspecified species with pneumonia	Pneumonia
A481	Legionnaires' disease	Pneumonia
B012	Varicella pneumonia	Pneumonia
B052	Measles complicated by pneumonia	Pneumonia
B0681	Rubella pneumonia	Pneumonia
B250	Cytomegaloviral pneumonitis	Pneumonia
J09X1	Influenza due to ident novel influenza A virus w pneumonia	Pneumonia
J1000	Flu due to oth ident flu virus w unsp type of pneumonia	Pneumonia
J1001	Flu due to oth ident flu virus w same oth ident flu virus pna	Pneumonia

© 2025 Molloy MJ et al. *JAMA Network Open*.

J1008	Influenza due to oth ident influenza virus w oth pneumonia	Pneumonia
J1100	Flu due to unidentified flu virus w unsp type of pneumonia	Pneumonia
J1108	Flu due to unidentified flu virus w specified pneumonia	Pneumonia
J120	Adenoviral pneumonia	Pneumonia
J121	Respiratory syncytial virus pneumonia	Pneumonia
J122	Parainfluenza virus pneumonia	Pneumonia
J123	Human metapneumovirus pneumonia	Pneumonia
J1281	Pneumonia due to SARS-associated coronavirus	Pneumonia
J1289	Other viral pneumonia	Pneumonia
J129	Viral pneumonia, unspecified	Pneumonia
J13	Pneumonia due to Streptococcus pneumoniae	Pneumonia
J14	Pneumonia due to Hemophilus influenzae	Pneumonia
J150	Pneumonia due to Klebsiella pneumoniae	Pneumonia
J151	Pneumonia due to Pseudomonas	Pneumonia
J1520	Pneumonia due to staphylococcus, unspecified	Pneumonia
J15211	Pneumonia due to methicillin suscep staph	Pneumonia
J15212	Pneumonia due to Methicillin resistant Staphylococcus aureus	Pneumonia
J1529	Pneumonia due to other staphylococcus	Pneumonia
J153	Pneumonia due to streptococcus, group B	Pneumonia
J154	Pneumonia due to other streptococci	Pneumonia
J155	Pneumonia due to Escherichia coli	Pneumonia
J156	Pneumonia due to other Gram-negative bacteria	Pneumonia
J157	Pneumonia due to Mycoplasma pneumoniae	Pneumonia
J158	Pneumonia due to other specified bacteria	Pneumonia
J159	Unspecified bacterial pneumonia	Pneumonia
J160	Chlamydial pneumonia	Pneumonia
J168	Pneumonia due to other specified infectious organisms	Pneumonia
J17	Pneumonia in diseases classified elsewhere	Pneumonia
J180	Bronchopneumonia, unspecified organism	Pneumonia
J181	Lobar pneumonia, unspecified organism	Pneumonia
J188	Other pneumonia, unspecified organism	Pneumonia
J189	Pneumonia, unspecified organism	Pneumonia
J851	Abscess of lung with pneumonia	Pneumonia
J80	Acute respiratory distress syndrome	Respiratory failure; insufficiency; arrest
J9600	Acute respiratory failure, unsp w hypoxia or hypercapnia	Respiratory failure; insufficiency; arrest
J9601	Acute respiratory failure with hypoxia	Respiratory failure; insufficiency; arrest
J9602	Acute respiratory failure with hypercapnia	Respiratory failure; insufficiency; arrest

© 2025 Molloy MJ et al. *JAMA Network Open*.

J9620	Acute and chr resp failure, unsp w hypoxia or hypercapnia	Respiratory failure; insufficiency; arrest
J9621	Acute and chronic respiratory failure with hypoxia	Respiratory failure; insufficiency; arrest
J9622	Acute and chronic respiratory failure with hypercapnia	Respiratory failure; insufficiency; arrest
J9690	Respiratory failure, unsp, unsp w hypoxia or hypercapnia	Respiratory failure; insufficiency; arrest
J9691	Respiratory failure, unspecified with hypoxia	Respiratory failure; insufficiency; arrest
J9692	Respiratory failure, unspecified with hypercapnia	Respiratory failure; insufficiency; arrest
R061	Stridor	Stridor
B340	Adenovirus infection, unspecified	Viral infection
B341	Enterovirus infection, unspecified	Viral infection
B342	Coronavirus infection, unspecified	Viral infection
B343	Parvovirus infection, unspecified	Viral infection
B344	Papovavirus infection, unspecified	Viral infection
B348	Other viral infections of unspecified site	Viral infection
B349	Viral infection, unspecified	Viral infection
B970	Adenovirus as the cause of diseases classified elsewhere	Viral infection
B9710	Unsp enterovirus as the cause of diseases classd elswhr	Viral infection
B9711	Coxsackievirus as the cause of diseases classified elsewhere	Viral infection
B9712	Echovirus as the cause of diseases classified elsewhere	Viral infection
B9719	Oth enterovirus as the cause of diseases classd elswhr	Viral infection
B9721	SARS-associated coronavirus causing diseases classd elswhr	Viral infection
B9729	Oth coronavirus as the cause of diseases classd elswhr	Viral infection
B974	Respiratory syncytial virus causing diseases classd elswhr	Viral infection
B975	Reovirus as the cause of diseases classified elsewhere	Viral infection
B976	Parvovirus as the cause of diseases classified elsewhere	Viral infection
B9781	Human metapneumovirus as the cause of diseases classd elswhr	Viral infection
B9789	Oth viral agents as the cause of diseases classd elswhr	Viral infection
J1282	Pneumonia due to coronavirus disease 2019	Viral Infection (COVID-19)
U071	COVID-19	Viral Infection (COVID-19)

© 2025 Molloy MJ et al. *JAMA Network Open*.

eTable 2. Respiratory Pathogen Testing Codes and Categories

CTC Codes	Test Description	Testing Category
364350	SARS-CoV-2 (COVID-19) test, unspecified	COVID-Only
364352	SARS-CoV-2 (COVID-19) antigen	COVID-Only
364355	Other specified SARS-CoV-2 (COVID-19) test	COVID-Only
382345	SARS-CoV-2 (COVID-19) molecular pathology testing, any method (DNA probe, PCR, RNA, RT-PCR)	COVID-Only
382726	Respiratory pathogen panel, 6-11 targets, molecular pathology testing, any method	Large Panels
382727	Respiratory pathogen panel, 12-25 targets, molecular pathology testing, any method	Large Panels
382728	Respiratory pathogen panel, unspecified # targets, molecular pathology testing, any method	Large Panels
382725	Respiratory pathogen panel, 3-5 targets, molecular pathology testing, any method	Targeted (≤ 5 Targets)
382350	Combo SARS-CoV-2 and Multiple Respiratory Viral Organism molecular pathology testing, any method (DNA probe, PCR, RNA, RT-PCR)	Targeted (≤ 5 Targets)
364152	Influenza A and B antigen	Targeted (≤ 5 Targets)
364153	Influenza A/H	Targeted (≤ 5 Targets)
364162	Parainfluenza antigen	Targeted (≤ 5 Targets)
364163	Metapneumovirus (hMPV)	Targeted (≤ 5 Targets)
364190	Adenovirus	Targeted (≤ 5 Targets)
364242	Respiratory syncytial virus antigen	Targeted (≤ 5 Targets)
382042	Microbial identification nucleic acid probe	Targeted (≤ 5 Targets)
382052	Microbial identification nucleic acid probe with amplification	Targeted (≤ 5 Targets)
382202	Common human coronavirus (229E, NL63, OC43, HKU1, etc.), molecular pathology testing, any method	Targeted (≤ 5 Targets)
382237	Influenza virus PCR	Targeted (≤ 5 Targets)
382238	Influenza virus RT-PCR	Targeted (≤ 5 Targets)
382239	Influenza RNA	Targeted (≤ 5 Targets)
382240	Influenza virus DNA probe	Targeted (≤ 5 Targets)
382245	Parainfluenza virus PCR	Targeted (≤ 5 Targets)
382246	Parainfluenza virus RT-PCR	Targeted (≤ 5 Targets)
382247	Parainfluenza virus RNA	Targeted (≤ 5 Targets)
382248	Parainfluenza DNA probe	Targeted (≤ 5 Targets)
382253	Metapneumovirus PCR	Targeted (≤ 5 Targets)
382254	Metapneumovirus RT-PCR	Targeted (≤ 5 Targets)
382255	Metapneumovirus RNA	Targeted (≤ 5 Targets)
382269	Adenovirus PCR	Targeted (≤ 5 Targets)
382270	Adenovirus RT-PCR	Targeted (≤ 5 Targets)
382271	Adenovirus RNA	Targeted (≤ 5 Targets)
382272	Adenovirus DNA probe	Targeted (≤ 5 Targets)
382317	Respiratory syncytial virus PCR	Targeted (≤ 5 Targets)
382318	Respiratory syncytial virus RT-PCR	Targeted (≤ 5 Targets)
382319	Respiratory syncytial virus RNA	Targeted (≤ 5 Targets)

© 2025 Molloy MJ et al. *JAMA Network Open*.

382320	Respiratory syncytial virus DNA probe	Targeted (≤ 5 Targets)
382325	Rhinovirus PCR	Targeted (≤ 5 Targets)
382326	Rhinovirus RT-PCR	Targeted (≤ 5 Targets)
382327	Rhinovirus RNA	Targeted (≤ 5 Targets)
382328	Rhinovirus DNA probe	Targeted (≤ 5 Targets)

© 2025 Molloy MJ et al. *JAMA Network Open*.
