# Supplementary material for: Trends in Respiratory Pathogen Testing at US Children’s Hospitals
Source: JAMA Netw Open. 2025 Mar 6;8(3):e250160. doi: 10.1001/jamanetworkopen.2025.0160 (PMC11886727; doi:10.1001/jamanetworkopen.2025.0160)
Supplement: Supplement 2. — Data Sharing Statement [file jamanetwopen-e250160-s002.pdf]

Data Sharing Statement

Molloy. Trends in Respiratory Pathogen Testing at US Children's Hospitals. *JAMA Netw Open*. Published March 06, 2025. doi:10.1001/jamanetworkopen.2025.0160

Data
Data available: No

Additional Information
Explanation for why data not available: Data from the Pediatric Health Information System is proprietary.
